# Sexual and reproductive health in Ethiopia: gains and reflections over the past two decades

**DOI:** 10.1186/s12978-022-01464-0

**Published:** 2022-08-09

**Authors:** Lisa M. DeMaria, Kimberly V. Smith, Yemane Berhane

**Affiliations:** 1 Mathematica, Inc., 600 Alexander Park, Suite 100, Princeton, NJ 08540 USA; 2 Epidemiology and Public Health, Addis Continental Institute of Public Health, P.O. Box 26751/1000, Addis Ababa, Ethiopia

## Introduction

Over the past two decades, through a series of concerted policies, programs, and commitments, Ethiopia has made notable advances in improving the reproductive health of its population, including expanding family planning (FP) information and services to larger segments of the population. Starting with the first Health Sector Development Plan in 1997, the Ethiopian government has invested heavily in health system strengthening and fostered a supportive policy environment for the expansion of access to health services and sexual and reproductive health (SRH) programming. The national health extension program and the accelerated expansion of primary health care services to increase both the availability and accessibility of essential services have both proven pivotal to expanding FP access, most notably among the country’s rural population. The government’s FP2020 commitments signaled its prioritization of increased funding for FP services and focus on adolescents and youth. The National Youth Policy enacted in 2004 and subsequent adolescent and youth (AY) SRH strategies—which expanded services to Ethiopia’s large youth population—provided a supportive AYSRH policy environment that has fostered improved reproductive health outcomes among this population. Similarly, the liberalization of the abortion law in 2005 expanded the conditions under which safe abortion care can be provided and expanded access to abortion care by authorizing midwives to provide abortion services.

These policies and programs have resulted in impressive gains. FP use has increased more than fivefold over the past two decades, with use of modern contraceptives rising from 6.6% among married women of reproductive age in 2000 to 40.5% in 2019. Over that same period, the total fertility rate dropped from 5.5 to 4.1 children per woman. Other notable changes included a decline in the maternal mortality ratio from 871 to 401 women per 100,000 live births between 2000 and 2017 [1, 2], in large part because of the liberalization of abortion. The median age at first marriage also rose from 16.0 years in 2000 to 17.1 in 2016 [3].^1^

Despite these remarkable achievements, however, coverage of reproductive health information and services remains low, with a large gap between current coverage rates and the universal health coverage (UHC) targets laid out in the country’s 2016–2020 Health Sector Transformation Plan (HSTP) [1, 4, 5]. More than one in five Ethiopian women still have an unmet need for FP; among adolescents, information on reproductive health is still largely shared through friends and is often inaccurate [3].

With the launch of FP2030 and less than a decade left for countries worldwide to meet their Sustainable Development Goals (SDGs) and the SDG UHC target, the coming years will require high quality information—a key pillar of the current HSTP—to inform the development and implementation of impactful SRH programs and initiatives to accelerate progress [6, 7]. Thus, we are at an opportune moment to pause, gather, and reflect on lessons learned around Ethiopia’s improvement efforts for FP, reproductive health, and adolescent health outcomes and identify what is required in the next decade to achieve the country’s goal to meet the SRH needs of its entire population.

This supplement endeavors to capture some of the key SRH lessons learned over the past two decades. It brings together the work and experience of researchers, practitioners, and policy makers engaged in FP and reproductive health work in Ethiopia. This supplement includes diverse perspectives, types of evidence, and insights on Ethiopia’s reproductive health context and the way forward. The supplement is structured around three main issue areas: (1) FP and contraceptive use, (2) AYSRH, and (3) abortion. First, the contributors present evidence on overall trends in FP and contraceptive use and examine determinants of use, both of which vary tremendously across regions and population groups. Getinet Yirtaw et al. identify important geographic differences in contraceptive ideation and future intention to use FP [8]. Other articles in this supplement provide insights into the sociocultural determinants of contraceptive use. Sedlander et al. explore determinants of misconceptions around contraceptive methods and provide insight into pathways to self-efficacy for FP use [9]. Smith et al. and Kapadia-Kundu et al. explore social and gender norms that influence FP use, highlighting the role of men’s support and equitable couples’ communication and decision making as key facilitators of FP use [10, 11].

The following section presents research focused on the adolescent and youth population—exploring the rapidly changing environment and social norms that affect their attitudes and behaviors and guide the debut and early stages of their reproductive lives. Akwara et al. review the progress made on adolescent reproductive health indicators over the past two decades [12]. Erulkar and Lindstrom et al. provide evidence on how these changing norms impact the ages of first marriage and first sex [13, 14]. Effective programming that reaches youth is critical. To that end, Chowdhary et al. explore the factors that influence the sustainability of a peer education program [15].

Finally, this supplement reflects on progress and remaining challenges in abortion care. Holcombe et al. provide a detailed analysis of the actors and processes that contributed to the reform of Ethiopia’s abortion law, culminating with its liberalization in 2005 [16]. Vernaelde et al. describe how the United States’ reinstatement and expansion in 2017 of the Global Gag Rule threatened Ethiopia’s national commitment to make abortion available [17]. Finally, social mores and opinions around abortion continue to present barriers to access, even when abortion is legal. Fekadu et al. and O’Connell et al. address stigma at the provider and community level, exploring barriers that this stigma creates to those seeking abortion care [18, 19].

Across the supplement’s compilation of articles, several common themes emerge. Demographic determinants related to FP use specifically, and women’s empowerment more broadly, point to remaining challenges to continued FP progress. Notably, national-level improvements in the modern contraceptive prevalence rate (mCPR) mask important geographic disparities, both urban–rural and across different regions of the country. Women in rural areas have an mCPR of 38.2% and tend to adopt an FP method later than their urban counterparts: their first use is after giving birth to an average of 2.2 children, compared to an average of 0.8 among women in urban areas [6, 20]. In addition, the four emerging regions (Afar, Benishangul-Gumu, Gambella, and Somali) have mCPRs below the national average, with Afar and Somali having particularly low rates of 12.7% and 3.4%, respectively [3].

Important gaps also remain in contraceptive and reproductive health knowledge and self-efficacy. Sociocultural barriers to contraception, AYSRH, and abortion information and services continue to be pervasive, with FP and reproductive health topics remaining taboo for much of the population.

## Looking to the future

In addition to the longstanding themes highlighted in this supplement, additional challenges to increasing access to and use of quality reproductive health services have emerged in recent years. Over the past two years, the COVID-19 pandemic has compromised access to reproductive health services in Ethiopia as well as globally [21–23]. The effects of curtailed access to FP services and disruptions in health care services more broadly due to COVID-19 have been documented. Studies comparing 2019 administrative data with 2020 and 2021 data find significant decreases in new contraceptive acceptors (−9.3%), safe abortion care users (−9.3%), and numbers of adolescents receiving contraceptive care (−3.5%) [24, 25]. Humanitarian crises, notably those caused by internal displacement and/or food insecurity, also threaten continued progress and important gains [26]. The Ethiopian government estimates that of the nearly 6 million people with humanitarian needs, 1.2 million are women and girls requiring FP and maternal health care [7]. The growing evidence of sexual and gender-based violence associated with the internal conflicts requires policy and programmatic interventions to ensure that victims receive timely care that meets their physical and mental health needs [27, 28].

Moving forward, several health policy areas should be prioritized to ensure continued progress toward national FP2030 and UHC goals and to achieve the SDGs by 2030. Strengthening service delivery—and, in particular, quality of care—remains a priority area to ensure continued advancement. Engaging the private sector in FP service delivery efforts and ensuring commodities supplies—both included in Ethiopia’s FP2030 commitments—will further strengthen and expand FP service delivery. Improving the quality of and equity in access to health care are top priorities under HSTP-II and a particular focal area highlighted by Kibret and Gebremedhin [29]. The quality of FP counseling, for example, remains low, with only 30% of women in a 2018 survey reporting that they received sufficient information during their counseling sessions with the service provider, a proportion that remains relatively unchanged from 2014 [30]. Socioeconomic factors play a role in who receives quality care, with women of low or no education and women in the poorest wealth quintiles reporting less and worse quality FP counseling [20]. As outlined in the government’s FP2030 commitments, innovation in the design and delivery of FP services should prioritize self-care, digital health, and advancing the role of the private sector in service provision [7].

The Reproductive Health Strategic Plan [31] and the AY health strategy [32]—both covering the 2021–2025 period and developed with input from a broad range of stakeholders from government, civil society, nongovernmental, and academic organizations—provide detailed pathways forward. The commentaries in this supplement highlight facilitators and barriers to achieving the goals of these strategic plans. Akwara et al. and Admassu et al. advocate for a broad policy framework, including expanding enabling laws, policies, and funding to ensure that quality SRH services are adequately extended  to reach the youth population [12, 33]. The current, and third, AY health strategy provides key parameters for advancing AY health: it sets key objectives and highlights leadership and accountability, including youth leadership and engagement, as critical strategies to meet the government’s FP2030 commitment to improve access to AYSRH-responsive services and to achieve better AYSRH outcomes [6, 33]. Feyssa and Gebru stress the need to continue to dismantle structural barriers to abortion services, such as fees for abortion care as well as stigma among providers, to ensure that abortion services are made widely available [34]. Overcoming these barriers is integral to achieving the government’s goal to ensure universal provision of safe abortion and post-abortion care across all the country’s health centers and hospitals [31]. The government’s FP2030 commitment to sustain and increase domestic financing of FP programs will be crucial to achieve the goals in these three areas of FP, AYSRH, and abortion care.

The design and implementation of SRH policies and programs must be supported by high quality evidence and data to ensure effectiveness and impact. Three priority areas for research are emerging. First is a clear and urgent need for implementation research on how to adapt and apply high impact practices (HIPs) to the Ethiopian context and specific populations. Much of the recent literature, including that contained in this supplement, focuses on determinants of FP use or access, nationally and within particular regions and populations. However, there is a wealth of information on HIPs to achieve FP success [35], and using implementation research frameworks can help test and adapt those HIPs to different national and subnational contexts [36]. A second, related need is for further research on how to shift sociocultural norms and, in turn, behaviors to ensure that women, youth, and families are able to identify and act on their FP and reproductive needs and preferences. For unmarried adolescents, for abortion, and for other service areas in which stigma and taboo are strong, shifting cultural norms is a particularly important need. Third, supporting service delivery efforts with rigorously designed and sufficiently funded impact and implementation evaluations will help identify what approaches are effective and provide insights on how to implement them to ensure and maximize their effectiveness in different contexts and potentially at scale. Finally, strengthening partnerships and collaboration among practitioners, policy makers, and researchers in the identification, design, and execution of research and evaluation efforts will help ensure the relevance, applicability, and actionability of findings that emerge from them.

## Conclusions

Over the past two decades (2000–2020), Ethiopia has built a foundation of laws, policies, and programs that support women’s right to high quality FP and reproductive health information and services. In turn, progress, while gradual, has been continuous and remarkable. Ensuring quality and equity in service provision efforts and fully addressing the needs of those in rural areas and youth remain challenges, and the COVID-19 pandemic and humanitarian crises pose serious risks to gains made to date in the country. Strong and data-driven implementation of current policies and programs, including those that support achievement of UHC and related SDG and FP2030 goals, will contribute significantly to ensuring continued gains in the decades to come.

## Data Availability

Not applicable.

## Abbreviations

AY: Adolescent and youth
FP: Family planning
HIP: High impact practices
HSTP: Health sector transformation plan
mCPR: Modern contraceptive prevalence rate
SDG: Sustainable development goals
SRH: Sexual and reproductive health
UHC: Universal health coverage

## Footnotes

1 Median age among women who were 25 to 49 years old at the time of the survey.
